# Innovation in Oncology Drug Development

**DOI:** 10.1155/2019/9683016

**Published:** 2019-11-23

**Authors:** Matthew Huber, Brian Huber

**Affiliations:** ^1^Department of Medicine, Georgetown University Medical Center, 4000 Reservoir Rd. NW, Washington, DC 20007, USA; ^2^ICON Plc, 79 T.W. Alexander Drive, 4401 Research Commons Bldg., Suite 300, Durham, NC 27709, USA

## Abstract

Significant progress has been made in our understanding of the molecular lesions responsible for tumor cells to exhibit uncontrolled growth while circumventing normal mechanisms of apoptosis and their ability to migrate and invade normal tissues while evading recognition and destruction by the immune system. This understanding has enabled the development of therapies specifically targeted to these lesions coupled to innovative treatment regimens to most effectively use these new targeted therapies with precision in selected subpopulations of patients. Innovation at the scientific and clinical levels has been appropriately embraced and supported at the FDA, resulting in regulatory innovation to facilitate and adapt to the Precision Medicine environment.

## 1. Introduction

Progress continues to be made in the treatment of cancer. In April 2018, the National Cancer Institute released its latest statistics on overall cancer deaths in the U.S. Cancer mortality is dropping at yearly rates of 1.8% for men, 1.4% for women, and 1.4% for children. Better prevention, earlier detection, and knowledge of the causative genetic lesions coupled to targeted therapies all play a part in this progress. This article highlights some recent, innovative changes that are occurring in oncology clinical drug development resulting from

1. Advances in the molecular understanding of cancer with associated targeted pharmacological interventions
2. The necessity to bring effective and valuable oncology drugs to patients more efficiently and with greater probability of success
3. The adaptability of the FDA to support clinical innovation based upon the evolution of the supporting science

What is the fuel that is driving innovation in oncology drug development?

### 1.1. Medical Necessity

Overthe last two decades, significant progress has been made in the treatment of some specific cancers, as measured by 5-year survival rates (Table 1) [1]. Testicular cancer is an excellent example of a success story in oncology. Testicular cancer is highly treatable and usually curable. If the cancer remains localized to the testis (Stage 1), it has almost a 100% cure rate in the US. This current clinical result is a vast improvement from the treatment response obtained in the 1970s, mainly due to emphasis on early detection via self-examination and the introduction and proper use of platin drugs.

Melanoma is another rapidly evolving success story. If caught at an early stage, the 5-year survival rate of melanoma can reach a remarkable 98%, but this drops to less than 20% if the disease is disseminated at diagnosis. The limited success story with melanoma and the continued optimism regarding improvements in clinical outcomes are the result of a combination of

- Heightened awareness for both prevention and early detection
- Deeper understanding of the molecular lesions associated with the cancer
- Introduction of B-Raf and MEK targeted therapies, such as vemurafenib, dabrafenib, and trametinib and their combination use [2]
- Introduction of recent immunotherapies, such as ipilimumab, pembrolizumab, and nivolumab

Despite specific success stories, other cancers remain recalcitrant to all current treatment regimens (Table 2 illustrates 8 examples of tumor types that have a 5-year survival rate of less than 30%). As such, there is a critical medical necessity to be able to bring better medicines to these patients more quickly and more effectively regarding time, cost, and probability of success.

### 1.2. Cost to Patients

From 2009 to 2014, the FDA gave marketing approval for 51 different oncology drugs in 63 indications [3]. The median cost to patients for these 51 new oncology drugs was over $100,000/year. Table 2 shows the cost of these drugs based upon the primary clinical endpoint used for initial approval. The important analysis of Mailankody and Prasad [3] illustrated that there was no significant relationship between yearly cost and the actual improvement in either progression free survival (PFS) or overall survival (OS). In addition, their analysis also illustrated that there was no significant price difference between novel mechanisms approved for the first time and “next-in-class” or “fast follower” drugs. Taken collectively, their analysis suggests that pricing in the US market may not be primarily driven by clinical value but rather simply by market forces. However, it must be realized that this is a relatively limited analysis over a limited time period.

The total cost associated with comprehensive cancer care in the US was approximately $125B in 2010, and this is expected to rise to $158B by 2020 [4]. The cost restricted to oncology products, excluding medical services, was approximately $42B in 2014. This was an approximate increase of 25% or $10B increase in just 4 years from 2010 to 2014 [5]. This cost trend is unsustainable. With the ever-growing pressures on oncology drug prices, it is imperative to innovate the oncology drug development process to be able to bring innovative medicines to patients faster and cheaper and with a higher probability of clinical success.

### 1.3. Cost of Development and Falling Productivity

The pharmaceutical industry, in general, is facing a perceived productivity crisis that is necessitating innovation [6]. As R&D spending has increased, the resulting “reward,” as defined as marketing approval for a new product, has declined. Estimating the perceived decline in productivity, it appears that for every 1 billion US dollars spent, there is a decrease by half the number of new drugs approved over each advancing decade [6].

New, innovative approaches are needed along the entire clinical drug development process in oncology to meet the challenges of today's medical environment. This is especially critical since it has been estimated that the current lifetime risk of developing cancer is 42% and 38% in men and women, respectively, in the US. [7].

## 2. Recent Innovation in Clinical Drug Development and Approval for Oncology Drugs

**Figure 1:**
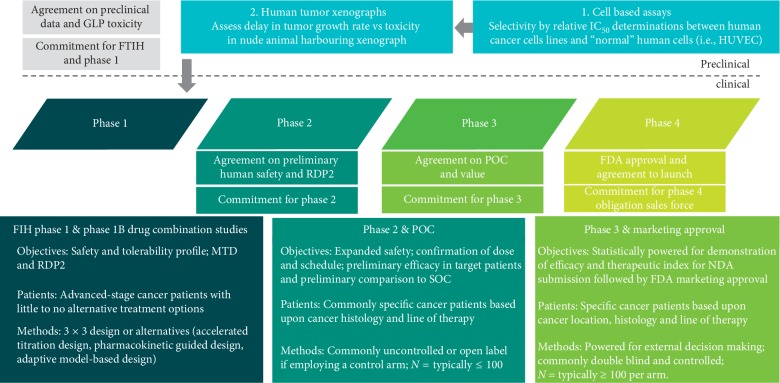
Typical Clinical Development Pathway for a Cytotoxic Cancer Chemotherapy Drug. Commonly used preclinical and clinical development pathway for cytotoxic chemotherapy drugs. A typical pathway for the development of cytotoxic cancer drugs started with demonstration of differential cytotoxicity in human cancer cell lines compared to nontransformed human umbilical cord endothelial cells (HUVECs). This was routinely followed by tumor xenograft studies in nude mice with nontoxic doses of the test drug (doses that did not produce weight loss) that are growing subcutaneous or orthotopic human tumor tumors. Efficacy was based upon slowing down of human tumor growth rate or even tumor regressions. This was followed by compliance with the regulatory requirements needed for approval of an IND to be able to treat patients in phase 1. Phase 1: the purposes of phase I studies for cytotoxic drugs are to assess the initial safety/tolerability and identify the maximal tolerated dose (MTD) and the recommended dose for phase 2 studies (RDP2). Phase I studies for cytotoxic cancer drugs are typically conducted in cancer patients since the drug is predicted to have safety concerns. Phase 2: with the identification of an acceptable dose/schedule from phase 1, supported by PK and even potentially PD data, it is possible to proceed to phase II exploratory therapeutic/efficacy trials in selected patient populations to get an initial estimate on antitumor efficacy in one or more tumor types while concomitantly expanding the safety data base on the investigational oncology compound. Phase 3: typically for registration and marketing approval. Commonly compares the overall safety and efficacy of the new treatment to the standard of care in a randomized, statistically rigorous, and blinded trial. However, it must be recognized, that by definition, these trials generate data from a highly controlled setting, which may not be reflective of “real-world” settings. Phase 4: phase 4 trials are also known as postmarketing surveillance trials. Phase 4 trials involve the safety surveillance or pharmacovigilance and ongoing technical support of a drug. Phase 4 trials generate additional important data that require longer periods of time or large patient populations to emerge, such as rare side effect profiles. These postapproval safety signals may result in the drug being withdrawn from the market or the label being more restrictive. Phase 4 studies may be required by regulatory authorities or may be initiated by the sponsoring company. MTD, maximal tolerated dose; PDP2, recommended dose for phase 2; HUVEC, human umbilical cord vascular endothelial cells; SOC, standard of care; NDA, new drug application.

Until the 1990's, the understanding of tumor biology was largely descriptive in nature—tumor cells divide faster than most normal cells, tumor cells are able to migrate and invade normal tissues, tumor cells are able to evade recognition and destruction by the immune system, and tumor cells appear to evade normal mechanisms of senescence and apoptosis. Based largely on these descriptive characteristics, cytotoxic drugs were developed that had modest positive clinical outcomes in specific tumor types despite exhibiting relatively narrow therapeutic indices. The paradigm for development of a typical cytotoxic drug are relatively standard (Figure 1).

Starting in the late 1980's, there was an explosion in our understanding of the molecular lesions that cause and maintain the transformed phenotype. These lesions enable deregulated cell growth, immune evasion, metastatic capability, and escape from apoptosis and senescence. This molecular understanding has resulted in the development of lesion-specific, targeted therapies linked with validated predictive biomarker test to identify tumors with these specific genetic lesions. These targeted therapies have changed the paradigm for oncology drug development and clinical use into a model of *Precision Medicine* [8, 9].

### 2.1. Personalized Medicine and Precision Medicine

#### 2.1.1. Personalized Medicine

**Figure 2:**
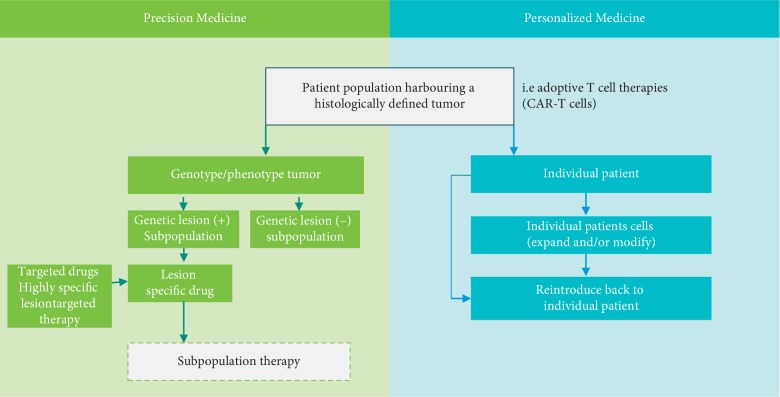
Precision Medicine verse Personalized Medicine. Personalized Medicine is defined as the creation of drugs that are unique to an individual patient and as the development of lesion-specific targeted drugs and the precise and selective use of those targeted therapies in specific subpopulations of patients whose tumors harbor those specific lesions.

It is not simple semantics to make a distinction between the terms *Personalized Medicine* and a similar term called *Precision Medicine*. There is a distinction between these terms despite sometimes being inappropriately used interchangeably (Figure 2). Personalized Medicine is more accurately defined as the creation of drugs or medical procedures that are unique to an individual patient. An example of a Personalized Medicine approach is adoptive T-cell therapies when an individual patient's cells are removed and ex vivo is modified to target the specific patient's tumor before being reintroduced back into the patient. One example of this is chimeric antigen receptor T cells (CAR-T cells) [10]. The scientific principle behind CAR-T cells is the ex vivo genetic alteration of T cells so that they can specifically target surface markers expressed in a patients' tumor. The T cells can be derived directly from the cancer patient (autologous T cells) or even derived from a healthy donor (allogenic T cells). Once obtained, these T cells are genetically modified to express a recombinant artificial T cell receptor combining T-cell activating functions and tumor-specific antigen binding domains that target tumor antigens which are specific to the patient's tumor. Once genetically modified, these cells may be expanded ex vivo and then reintroduced back to the patient harboring that tumor. Once reintroduced back to the patient, these genetically modified CAR-T cells can recognize the tumor-specific surface antigens, become activated, expand in vivo, and effectively function as cytotoxic T cells attaching the patient-specific tumor.

#### 2.1.2. Precision Medicine

This medical model is centered on appropriate diagnostic testing to genotype and/or phenotype tumors to enable more customized selection of drug treatments for specific subpopulations of patients. Hence, Precision Medicine relates to the development of lesion-specific targeted drugs and the precise and selective use of those targeted therapies in specific subpopulations of patients whose tumors harbor those specific lesions. The additional and corollary value of this approach is the capability NOT to use these targeted drugs in other subpopulations that do not have the specific lesion, since these subpopulations will likely experience little to no clinical benefit. Of course, this is NOT a new medical concept, but the ability to genotype/phenotype tumors to identify molecular lesions and then treat with specific targeted therapies has greatly advanced over the last 20 years. One of the first examples of Precision Medicine in oncology can be traced to the discovery and focused clinical development of imatinib (see Section 3.2.5) and rituximab. It is now firmly established the value of a precision medicine approach. In a meta-analysis of 570 single-arm phase-2 trials enrolling over 32,000 subjects, Schwaederle et al. [9] demonstrated that subpopulations who received targeted agents based upon the expression of predictive biomarkers had better ORR and PFS compared to those nonselected patients in which targeted therapies were used in a nonselective fashion or patients receiving cytotoxic agents. The superiority of a precision medicine strategy was also confirmed in a meta-analysis of pivotal oncology trials used for FDA approval [11].

However, simple descriptions of a precision medicine approach run the risk of oversimplifying the inherent complexity. It is well recognized that there is tremendous tumor heterogeneity within a patient's tumor. Tumor phenotypes evolve over time due to both selective pressures and genetic instability. Most importantly, treatment strategy is evolving from a binary approach (i.e., identification of a lesion = selection of a targeted therapy) to a computer-assisted (AI) approach that tries to address the multiple interacting genetic lesions with the multiple available targeted therapies and monitors this as it changes in response to drug treatment resulting in the emergence of new subpopulations of tumor cells.

**Figure 3:**
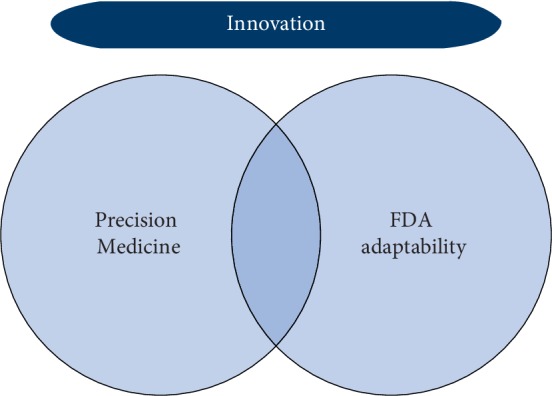
Innovation = Precision Medicine + FDA Adaptability. Innovation in cancer drug development has resulted from both the maturation of Precision Medicine and the forward thinking and flexibility of the FDA to recognize that targeted therapies require innovative targeted trials.

The maturation of Precision Medicine coupled to the adaptability and flexibility of the FDA has enabled significant innovation and profound changes in the clinical development, regulatory approval, and reimbursement practices in oncology (Figure 3), as exemplified in the remaining sections of this article.

### 2.2. Use of Phase 0

Phase 0, also known as microdosing studies, is a recent designation for optional exploratory trials to rapidly access whether an investigational drug has similar PK characteristics in human subjects as anticipated from preclinical studies. Phase 0 may be particularly valuable when the sponsor has some evidence to suggest that animal models may not be predictive of human PK parameter. Distinctive features of phase 0 trials include the following: being performed prior to traditional phase I trials; administration of single subtherapeutic doses to a small number of subjects to gather preliminary data on the drugs' pharmacodynamic and pharmacokinetic properties [12]. It was hoped that phase 0 studies could inform and expedite the development of promising oncology drugs by providing key PK and surrogate marker data for early decision making.

A phase 0 study provides little data on safety or efficacy, being by definition at a dose too low to cause any therapeutic effect. Phase 0 studies can however help prioritize multiple-drug candidates based upon pharmacokinetic parameters in humans, thereby helping to eliminate less-promising compounds very early and cheaply. Preliminary information can also be obtained on target interactions and surrogate markers, if these effects can be seen at subtherapeutic doses. In addition, phase 0 studies may help drive efficiency with more informed phase 1 starting doses. Since an estimated 40% of drugs fail in phase I trials because of unsuitable pharmacokinetics [13], the effective use of Phase 0 studies can enable rapid go/no-go development decisions based on relevant human data instead of relying on sometimes nonpredictive animal data.

The results of one of the first phase 0 trials established favorable biochemical and pharmacokinetic properties of the experimental drug ABT-888, a poly ADP-ribose polymerase (PARP) inhibitor, in 13 patients with advanced cancers. A critical path development parameter for ABT-888 was to identify a safe and tolerable ABT-888 dose that inhibits PARP, rather than identifying the MTD of ABT-888. ABT-888 was administered as a single oral dose of 10, 25, or 50 mg to evaluate ABT-888 PK parameters but also to examine PARP inhibition in tumor biopsy samples and PBMCs. The study very efficiently provided significant information regarding both PK and dose/schedule needed for the inhibition of PARP. This phase 0 very rapidly enabled subsequent studies using ABT-888 in combination with DNA damaging agents [14].

Clinicaltrials.gov has references to 5664 phase 0 trials in oncology. Despite this large number, it must be emphasized that the use of phase 0 trials is relatively limited [15]. To be tested in phase 0, an experimental drug should exhibit a wide therapeutic index in preclinical toxicology studies. Furthermore, ethical issues have been raised since phase 0 volunteers have virtually no chance of potential clinical benefit due to the subtherapeutic doses [16].

### 2.3. Innovation in Phase 1

#### 2.3.1. Dose Escalations

The clinical drug development paradigm for anticancer drugs has historically followed the very traditional “phased” approach with sequential, stand-alone trials, utilizing increasing numbers of patients exposed to the investigational drug to fulfill the objectives of that particular phase in development (Figure 1).

For cytotoxic drugs, phase 1 objectives are predominantly focused on identifying the maximal tolerated dose (MTD) and the recommended phase 2 dose (RP2D) appropriate for the majority of patients, either alone or in combination with other drugs. For nonselective cytotoxic drugs, the vast majority of phase 1 studies are conducted without regard to solid tumor specificity in cancer patients that have little to no alternative treatment options. In addition, these trials are typically single-arm, open label in relatively few patients. However, phase 1 trials are far from simple, having multiple interrelated components, such as patient selection, starting dose, dose increments/escalations, patients per dose level, definitions for maximal tolerated dose, and definitions for RP2D [17].

Multiple methodologies have been developed for phase 1 dose escalations for cytotoxic drugs—all being focused on 3 similar objectives and 1 similar assumption:

 Objectives:
  (1) Minimizing exposure of patients to both unsafe doses and subtherapeutic drug doses
  (2) Defining the recommended phase 2 dose (RP2D) and the maximal tolerated dose (MTD)
  (3) Identifing dose-limiting toxicities and determining if animal toxicity models are predictive of human toxicity
 Assumption:
  (1) Toxicity and efficacy increase with dose, typically in a sigmoidal dose-response curve; therefore, it is possible to identify a minimal and maximal toxic dose and effective dose

Phase 1 methodology falls into 2 broad designs: rule-based designs (i.e., 3 + 3 design) and model-based designs (i.e., continuous reassessment model). Within each broad phase 1 design, there are multiple methodologies that can be employed. These methodologies are described in detail in an Electronic Supplement to this paper (REF). Many phase 1 studies now use a combination of these methodologies by initiating studies with either a 3 + 3 or accelerated titration design that transits into a continuous reassessment model. This combined methodology may yield a more accurate and defined MTD and RP2D and has both the robustness of a rule-based approach with the flexibility of a continuous reassessment approach [18].

### 2.4. Evolution of Phase 1 Designs with Targeted Therapies

The development of specific targeted therapies and immunotherapies has necessitated the evolution of phase 1 objectives:

- Phase 1 patients are now more rigorously selected from subpopulations of patients whose tumors may positively respond to the targeted therapy.
- The clinical objective is not necessarily identifying the maximal tolerated dose, but rather a pharmacologically active dose where the biological target is appropriately affected. In fact, efficacy may occur at dose levels that do not produce clinically significant toxicity. Hence, using alternative phase 1 endpoints other than toxicity, such as pharmacodynamic endpoints of target inhibition, may be more appropriate for targeted therapies [19]. The pragmatic challenge of this approach is the necessity for robust supportive scientific data, availability of patient tissue to assess target inhibition, and a quantitative and validated target assay.

#### 2.4.1. Targeted Therapy and Expansion Cohorts

With the development of molecularly targeted agents, subpopulations of patients in early phase 1 studies can be specifically selected to increase the probability that they may receive clinical benefit. The result of which is that preliminary antitumor efficacy is now more likely to be determined in some phase 1 studies in those patients treated near efficacious dose levels. Due to this, it is now common that phase 1 protocols utilize expansion cohorts (EC) [20]. This has evolved to phase 1 trials for molecularly targeted agents commonly having 2 objectives combined in a single protocol:

1. A dose escalation portion to identify the RP2D
2. An expansion cohort phase where specific patients are tested to obtain additional PK data, evaluation of predative biomarkers, preliminary data on proof of mechanism and antitumor efficacy, and study objectives typically found in later-phase trials

A good example of this strategy was the clinical development of the anti-PD-1 MAB, Nivolumab. In the phase 1 study of nivolumab, ECs of patients with colorectal carcinoma (*N* = 16), melanoma (*N* = 16), renal cell carcinoma (*N* = 16), prostate cancer (*N* = 16), and non-small-cell lung cancer (*N* = 16) were utilized [21].

As such, phase I ECs are being effectively used more commonly in FIH studies to help accelerate the oncology drug development process [22]. Preliminary efficacy data obtained in phase 1 ECs can enable subsequent protocols to be more robust without incurring inappropriate risk. ECs are consistent with the goals and concepts described by FDA's expedited programs for serious conditions. It is now common to find FIH oncology trails with over 100 patients, and even a FIH phase 1 trial with a final total of 8 protocol amendments enrolling over 1,000 patients (below). FIH studies that utilize EC increased by >200% from 2006 (12% utilization) to 2011 (38% utilization) [22]. Between 2010 and 2015, 3% of all INDs submitted to the FDA Office of Hematology and Oncology Products employed ECs of greater than 100 patients [23]. The majority of these had efficacy as the clinical objective of the EC to be able to provide both sufficient safety and efficacy data for accelerated approval. However, it was also found that the majority of these trials failed to provide a robust statistical rationale for the size of the EC [23, 24], which may ultimately compromise the goal for accelerated approval.

**Figure 4:**
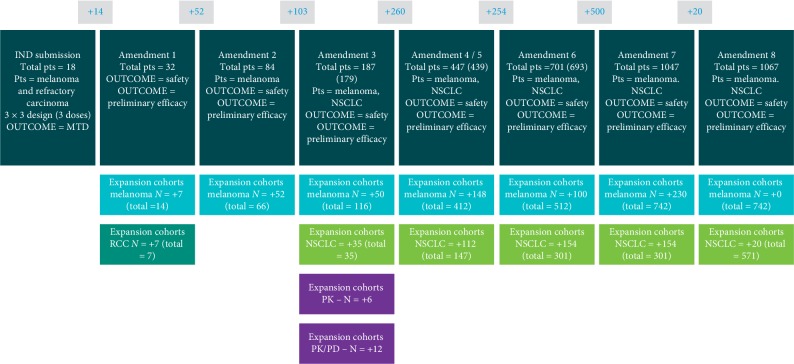
FIH Protocol (PN001) for pembrolizumab—*Multiple Expansion Cohorts.* Illustration Merck FIH pembrolizumab clinical development program. The initial IND submitted in 2010 was to enroll 18 patients with melanoma plus 14 additional patients in an EC with melanoma and renal cell cancer. Over the next 2.5 years, 8 protocol amendments were filed, enabling the FIH study to be expanded to 9 distinct ECs enrolling a planned 1,100 patients [21–23]. Successful implementation of this strategy resulted in accelerated approval for refractory, unresectable or metastatic melanoma and was supportive for approval in NSCLC.

An extraordinary example of the effective use of EC in FIH studies is the Merck FIH pembrolizumab clinical development program. The initial IND submitted in 2010 for pembrolizumab was to enroll 18 patients with melanoma plus 14 additional patients in an EC with melanoma and renal cell cancer. Over the next 2.5 years, 8 protocol amendments were filed, enabling the FIH study to be expanded to 9 distinct ECs enrolling a planned 1,100 patients [23] (Figure 4). As data demonstrating significant antitumor efficacy accumulated in this FIH study, amendments were directed at incorporating robust efficacy endpoints traditionally used in phase II/III studies.

With the use of Phase 1EC and seamless clinical designs (see Section 2.5), the terms phase 1, phase 2, and phase 3 are evolving from very distinct and separate stages into a less restrictive and seamless continuum of studies that are “*fit for purpose*” [25]. What is important to note is the continued strict ethical practice of careful progressive exposure of more and more subjects only when clinical risk/benefit warrants. The use of EC not only requires robust risk benefit analysis but also requires modifications of clinical conduct more aligned with the later-stage trails for potential accelerated approval (i.e., related to data capture, clinical monitoring, etc.). The effective use of early-stage ECs can expedite the development and accelerated approval of important medicines. The terminology “phase 1 expansion cohorts” can be somewhat misleading, since the clinical conduct utilized in these expansion cohorts can be more aligned with later-stage phases 2 and 3 trial methodologies.

Important shortcomings of phase 1 expansion cohorts are that a significant number of protocols, to date, have little statistical justification for the patients included in the expansion cohort. Even more importantly, many of these studies are not randomized, relying on historical comparator data with the associated risks and biases of any nonrandomized trial.

### 2.5. Seamless and Adaptive Trial Design in Oncology

There is a strong trend in oncology drug development that distinct demarcations between defined clinical development phases are being replaced by a seamless continuum of development. The benefits of evolving from a traditional clinical development approach characterized by sequential distinct phases of phase 1, 2, and 3 to a more integrated and seamless “fit for purpose” approach using adaptive design tools may greatly affect cost, time, and probability of success [25].

The traditional clinical development paradigm of conducting distinct phase 1, phase 2, and phase 3 studies before submission of an NDA could add years of “white space” to the development timeline.

Clearly, the clinical objective of seamless designs is not to diminish the appropriate independent IRB and FDA review of study conduct. The progressive exposure of more patients to an experimental drug is based upon the delicate balance between risk/benefit, and this concept is especially relevant and robustly applied to protocols utilizing a seamless deign. Many seamless design protocols include either a blinded or nonblinded interim analysis conducted by an independent review board. Prospectively defined in the protocol, the independent interim analysis must meet or exceed predefined goals for safety and efficacy before seamlessly enrolling additional patients.

The scientific advances that have enabled Precision Medicine have also fostered the innovative use of adaptive trail design and application of Bayesian statistics. Adaptive trail design uses accumulating data from the ongoing trail to modify certain aspects of the ongoing trail without undermining the validity and integrity of the trail. The potential adaptations for modifications are prospectively planned and designed to maintain statistical robustness and integrity, so a protocol amendment is unnecessary. Adaptive study designs are an important innovative advance in the clinical trial process.

Traditional clinical studies have prespecified treatment arms in which patients receive a predetermined therapy for a fixed period of time. Traditional clinical trials are rigidly designed to follow a specific protocol and do not take into consideration new scientific understanding and discovery as the trail progresses. With an adaptive study design, researchers can see how patients are responding to treatments, whilst the study is running and can alter aspects of the study, such as the compounds being investigated, adding additional cohorts, or even altering patient numbers. Hence, adaptive trial design gives the investigator greater flexibility to make modifications based upon the real-time learning as the trail progresses. All of these allow more patients to be randomized to the optimal treatment regimens and allows clinical conclusions to be determined more quickly and efficiently.

The types and uses of adaptive trail design are many and have permeated into all stages of clinical development, such as

1. Adaptive dose finding—often used in early stage development to more rapidly identify the minimally effective dose or the maximal tolerated dose. This can be accomplished by using the accumulated data to differentially expand certain cohorts compared to others.
2. Group sequential design—enables early stopping due to safety, futility or efficacy.
3. Sample size re-estimation—enables sample size adjustments or re-estimation based upon observed data at a defined interim timepoint in either a blinded or unblinded fashion and enables readjustment of sample size based upon the projected *P* value at the end of the study if this has been appropriately prospectively planned.
4. Adaptive seamless phase II/III design—a single study that combines both phase 2 objectives and phase 3 objectives where observations in phase 2 can be adapted, expanded, and confirmed in phase 3.
5. Biomarker-based adaptive design—enables adaptions based upon observed relationships between marker expression and an observed clinical outcome to enrich for patient subpopulations in the ongoing clinical trial.

There are many benefits to innovative adaptive clinical trial design, if employed correctly to maintain integrity and validity. Some of these benefits are as follows:

1. More ethical treatment of subjects in the trial by decreasing randomization to ineffective or toxic cohorts while increasing randomization to effective cohorts.
2. Flexibility if appropriately planned.
3. Based upon real-time trial data, the trail can be adapted to increase the optimal trial design to generate the correct answers, thereby increasing the overall probability of success.

## 3. Potential Cost Savings

### 3.1. Further Innovation in Trial Design: Umbrella and Basket Trials

As stated above, phase 1 oncology trials with nonspecific cytotoxic drugs have historically been conducted without regard to solid tumor histology in cancer patients that have little to no alternative treatment options. Then phase 2 and 3 oncology trials were histopathologic focused usually in a randomized trial comparing the experimental drug to what was currently the approved standard of care [26]. This process approaches the entire patient population as an average—average genetic profile, average metabolic profile, and average intermediary metabolism profile. This approach is suboptimal for targeted agents.

Now, each cancer histology can be subdivided into subpopulations of patients defined by specific genetic lesions (i.e., genetic signature), which subsequently dictates the specific targeted therapy to be utilized. The appreciation that cancer histologies are composed of patient subpopulations related to specific genetic lesions has generated two key observations:

1. A specific cancer histology can be subdivided into subpopulations of patients, each defined by a genetic signature. The genetic signature then indicates what specific targeted therapy should be utilized in that subpopulation of patients. Hence, different targeted agents will be rationally chosen to treat subpopulations of patients all with the same tumor histology.
2. Very different cancer histologies may possess subpopulations of patients that have a similar genetic signature. These subpopulations with common genetic signatures can occur in different percentages among the different tumor histologies. Hence, tumors with different histologies may contain subpopulations of patients with a common genetic signature that will respond to a similar targeted agent.

It is recognized as impractical to conduct separate clinical trials in all subpopulations of patients found in different histologies. This is compounded by the fact that, in some histologies, the genetic subpopulations may represent a very small number of patients. There is now a shift from histology-based trial design to genetic signature/biomarker-based trial design. Hence, treatment with a targeted agent is restricted to patients that are positive for the biomarker associated with the mechanism of that targeted therapy. This of course requires a robust, validated marker assay that can be readily utilized in a clinical setting.

**Figure 5:**
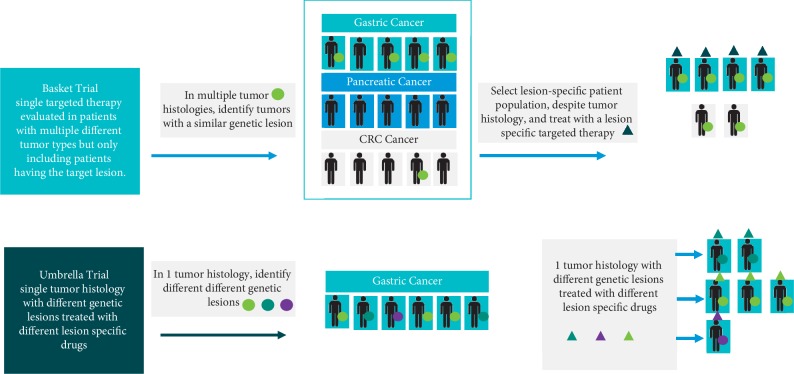
Basket and umbrella trials. Basket trial: single targeted therapy evaluated in patients with multiple different tumor types but only including patients having the target lesion. Umbrella trial: single tumor histology with different genetic lesions treated with different lesion-specific drugs.

It is now well recognized that targeted drugs require innovative targeted trials—two relatively new clinical trial strategies are the basket and umbrella study designs (Figure 5). The importance of these trial designs is that targeted treatments can be evaluated more efficiently and, most importantly, patients expressing a rare genetic signature for that tumor histology have much greater access to innovative targeted therapy [27].

#### 3.1.1. Basket Trials

Basket trials select patients into the trial based upon their tumor's genetic signature, regardless of the tumor histology. These studies will test the effect of a targeted drug in a variety of cancer histologies, provided that all the tumors express the specific marker. It is also recognized that the different tumor histologies enrolled in a basket trail will likely not respond identically to the targeted drug, despite all being positive for the marker. This is reflective of other characteristics of the tumor, such as intermediary metabolism, complex interactions with other genetic lesions, and the tumor milieu. While only evaluating one investigational targeted agent, the clinical evaluation is done simultaneously in several tumor histologies. An example of a basket trial is the BRAF V600 Vemurafenib study [28]. This study enrolled 122 patients, all expressing a BRAF V600 mutation in 5 distinct prespecified histology baskets.

#### 3.1.2. Umbrella Trials

An alternative innovative clinical approach for targeted therapies is that of an umbrella trial. In contrast to a basket trial, the umbrella trial evaluates multiple targeted therapies in relevant subpopulations of patients within a single histology. These trials allow investigators to evaluate multiple targeted agents in multiple subpopulations in a specific tumor histology in one clinical study. While a basket trail involves a single targeted therapy with associated single marker in multiple tumor histologies, an umbrella trial involves multiple targeted therapies with associated multiple markers in a single tumor histology.

#### 3.1.3. Super Umbrella Trials

The combination of a basket trial and an umbrella trial creates a “Super Umbrella Trial.” The design is the same as an umbrella trial, but open to multiple histologies, similar to a basket trail. An example is the NCI-MATCH study [29]. The NCI-MATCH study, originally opened in Fall 2015 with 10 treatment options, has subsequently been adapted to now having 30 active treatments. This study is open to solid tumor or lymphoma patients who progressed on standard therapy, with a plan to screen 6000 patients. Each treatment is an independent single-arm treatment with objective response rate as the primary outcome. Each treatment option has a set of rules mapping the biomarker and clinical information into a list of eligible treatments [29].

Two positive aspects of the basket and umbrella trail designs are as follows:

1. More efficient than evaluating multiple histologies separately, especially if the genetic signature is relatively rare in a particular histology
2. Patients expressing a rare genetic signature for a particular tumor or a rare tumor histology have much greater access to innovative targeted therapies

However, basket and umbrella trials, as currently designed, also have shortcomings:

1. Most basket and umbrella trials, to date, do not include a SOC arm for each histology. So historical control data are used to demonstrate superior clinical activity over SOC
2. Some arms may have a very small sample sizes if the mutation signature is rare for that histology
3. Most studies require a study-specific biomarker assay for eligibility
4. By design, basket and umbrella trails provide no learning regarding biomarker negative patients

### 3.2. FDA Programs to Expedite the Development, Review, and Potential Approval of Oncology Drugs

Innovation in oncology drug development is also occurring at the FDA. The FDA created four programs to expedite the development, review, and approval of drugs and biologics. The *Accelerated Approval, Priority Review, Fast Track,* and *Breakthrough Therapy* designations are programs for therapies intended “to address significant unmet medical need in the treatment of a serious or life-threatening condition” [30].

#### 3.2.1. Accelerated Approval

Created by the FDA in 1992, *Accelerated Approval* is intended to provide cancer patients early access to drugs that demonstrate sufficient preliminary activity. Accelerated approval utilize surrogate clinical endpoints that are thought to predict definitive clinical benefit. Surrogate endpoints in oncology studies, such as *objective response rate* (ORR) and *progression free survival* (PFS), typically require less time to assess and are thought to predict the more typical phase 3 endpoint of *overall survival* (OS). In 2012, the congress passed the Food and Drug Administration Safety Innovations Act, which confirms the FDA may use surrogate and intermediate clinical endpoints as a basis for therapy approval. Accelerated approval is usually contingent upon the drug sponsor conducting subsequent confirmatory trial(s) in a timely manner to more rigorously demonstrate that the drug is indeed associated with clinical benefit in the form of OS. If the subsequent trial(s) fail to demonstrate OS clinical benefit or if the sponsor does not demonstrate appropriate “due diligence” in performing the trial(s), the FDA could remove the drug from the market. The accelerated approval program provides patient access to promising new drugs based upon provocative preliminary safety and efficacy data, as the sponsor concurrently conducts robust confirmatory clinical trials using registration-appropriate clinical endpoints. *Accelerated Approval* regulations are especially relevant and helpful for deadly cancers that progress slowly.

The accelerated approval development strategy should be prospectively discussed with the FDA for early endorsement of the strategy, endpoints, and study details. Pembrolizumab (marketed as Keytruda) is an example of a biologic which received approvals for multiple new indications in 2018 through the *Accelerated Approval* pathway [31].

#### 3.2.2. Priority Review

The 1992 Prescription Drug User Act created the designations of *Priority Review*. Under the *Priority Review* designation, FDA resources are allocated with the intention of reviewing an NDA or BLA within 6 months [30]. Drugs with evidence of increased safety or efficacy in the treatment, diagnosis, or prevention of serious conditions can receive priority review status [30]. This includes drugs with evidence of limiting the adverse effects of current therapies, the ability to treat patients that have failed or cannot tolerate available therapies, or the ability to be used effectively in combination with available therapies. In the *Priority Review* process, the FDA is to assess the designation of *Priority Review* within 60 days of receiving an NDA or BLA, where the drug sponsors requested *Priority Review* status [30].

As one example—Larotrectinib, marketed as Vitrakvi, was approved under *Priority Review* in 2018 as the second ever tissue-agnostic cancer treatment [32, 33]. Larotrectinib therapy is approved for the treatment of patients with unresectable or metastatic solid tumors with neurotrophic receptor tyrosine kinase gene fusions and is an example of a drug which showed evidence of safety and effectiveness in a new subpopulation [30].

#### 3.2.3. Fast Track

The *Fast Track* designation can be utilized by the FDA for therapies which demonstrate a potential to treat serious conditions and fill unmet medical needs. *Fast Track* designation is reserved for conditions with no available therapy or for drugs which show the potential to improve the efficacy of available therapies [30]. Under the *Fast Track* designation, drug sponsors are provided the opportunity for increased meetings with the FDA throughout the clinical development process to ensure alignment on study design, data required for approval, use of biomarkers, and other concerns to help expedite drug development and review. *Fast Track* designation may be requested by a drug sponsor with the Investigational New Drug (IND) application but no later than the pre-NDA or pre-BLA meetings and should be supported by evidence that demonstrates a potential to fill unmet medical needs [30]. *Fast Track*-designated drugs may also meet the criteria for *Accelerated Approval* and *Priority Review.*

As one example, Abemaciclib, marketed as Verzenio, is a drug approved in 2018 under the *Fast Track* designation. Abemaciclib is approved in combination with an aromatase inhibitor for the treatment of hormone receptor-positive, human epidermal growth factor receptor 2-negative, advanced or metastatic breast cancer in postmenopausal women.

#### 3.2.4. Breakthrough Therapy

The *Breakthrough Therapy* designation can be utilized by the FDA for new therapies that treat a serious condition for which preliminary clinical evidence shows a substantial improvement over available therapies on clinically significant endpoints [30]. The *Breakthrough Therapy* designation is reserved for drugs that have clinical evidence demonstrating a clear advantage over available therapies, but that is generally not sufficient yet for full drug approval. While the *Fast Track* designation requires that a drug shows evidence for the potential to improve upon available therapies, the *Breakthrough Therapy* designation is held to the higher standard of showing preliminary clinical evidence of improving clinically significant endpoints. The evidence for the designation assessment may include surrogate or intermediate endpoints, similar to *Accelerated Approval*. In addition, the designation may include drugs with similar efficacy to available therapies and that shows evidence of improved safety. Under the *Breakthrough Therapy* designation, drug sponsors are provided all *Fast Track* features, guidance on drug development as early as phase 1, involvement of FDA senior level managers, *Rolling Review*, and other services to expedite review. *Breakthrough Therapy* designation may be requested by a drug sponsor as soon as they have sufficient clinical evidence to support this designation. Applications are typically submitted as amendments to an IND application and prior to submitting an NDA or BLA. *Breakthrough Therapy*-designated drugs may also be eligible to receive *Accelerated Approval* and *Priority Review* designations during the FDA review process [30]. Lorlatinib, marketed as Lorbrena, was approved in 2018 under the *Breakthrough Therapy* designation for the treatment of a specific form of non-small-cell lung cancer [34]. In addition, Keytruda, Vitrakvi, and Verzenio all received FDA approvals for certain indications in 2018 under the *Breakthrough Therapy* designation [34].

#### 3.2.5. Orphan Drug Act and Development of Drugs for Rare Cancers

In 1983, the congress established assistance for drugs that treat rare diseases (called orphan indications) including rare forms of cancers [35]. An orphan indication is defined as follows:

- Indication affecting fewer than 200,000 persons in the US. It is important to note that orphan indications INCLUDE diseases that may be very common, if not endemic, elsewhere in the world, but very rare in the US, such as many tropical diseases.
- a drug that will not be profitable within 7 years following FDA approval.

Orphan designation does not alter the standard regulatory requirements and processes for obtaining FDA marketing approval, although some statistical burdens may be pragmatically considered due to the very rare patient populations. However, orphan designation does qualify the drug sponsor for various development incentives, such as

1. FDA granting 7-year marketing exclusivity
2. Tax credits up to 50% of clinical development costs
3. Exemption/waiver of application fees

It is important to note how innovative, flexible, and data driven the FDA is on bringing clinically valuable cancer medicines to patients as quickly and safely as reasonably possible. It is very informative to examine three case studies that illustrate the evolution of the FDA review process. These case studies were expertly summarized in editorials by Chabner [36, 37].

*(1) Imatinib*. Chronic myelogenous leukemia (CML), also known as chronic granulocytic leukemia (CGL), is a disorder of bone marrow stem cells, characterized by proliferation of granulocytes and their precursor cells. CML is characterized by a specific reciprocal chromosomal translocation, involving chromosomes 9 and 22. This reciprocal translocation creates a BRC-ABL gene fusion, with the BRC gene component coming from chromosome 22 and the ABL gene component coming from chromosome 9. The chromosomal abnormality was first discovered by Hungerford and Nowell in 1959, [38, 39] and the specific translocation further defined by Rowley in 1973 [40] and is called the Philadelphia chromosome or Philadelphia translocation *t* (9; 22). The aberrant BCR-ABL fusion protein is a “deregulated” kinase, being constitutively “on,” resulting in uncontrolled cell division. Most importantly, this specific chromosomal translocation is found in 95% of CML cases.

Imatinib (Gleevec), a tyrosine kinase inhibitor, is selective for the inhibition of the kinase activity of the BCR-ABL fusion protein [41–43]. Imatinib is a true success story of both rational drug design and Precision Medicine. Imatinib was discovered by screening chemical libraries for inhibitory activity specific for the fusion protein. Pertinent for this discussion, the FDA granted accelerated approval for imatinib on May 10, 2001, for the treatment of CML, based upon three phase 2, open-label, and single-arm studies. The accelerated approval was conditional for the sponsor to conduct and submit a randomized phase III study. This is one of the first examples for conditional approval to be granted based upon an open-label, single-arm phase 2 results, and using Chambner's analogy, equivalent to running the 4-minute mile [36, 37].

*(2) Crizotinib*. The crizotinib clinical development strategy is very similar to that of imatinib. About 4% of patients with NSCLC have a specific chromosomal rearrangement that generates a tumor-specific fusion gene between the *EML4* gene (echinoderm microtubule-associated protein-like 4′) and the ALK gene (anaplastic lymphoma kinase). This tumor-specific fusion protein exhibits constitutive kinase activity promoting tumor growth. Crizotinib selectively inhibits the kinase activity of this tumor-specific fusion protein, The FDA granted accelerated approval for crizotinib in August 2011 for the treatment of EML4-ALK + NSCLC based upon a very positive phase I trial with two confirmatory single-arm phase 2 trials with a combined total of 255 patients [44].

*(3) Ceritinib*. Ceritinib is another drug that specifically targets NSCLC that is positive for ALK gene rearrangements. Accelerated approved was granted by the FDA in April 2014 for ALK-positive metastatic NSCLC who have either progressed on crizotinib or are intolerant to crizotinib. Hence, this is another treatment option for Alk-positive NSCLC patients that became “resistant” to or are intolerant of crizotinib. Accelerated approval was based ORR in a single-arm, open-label, phase 1 clinical trial of 163 Al -positive NSCLC patients that became “resistant” or are intolerant to crizotinib. This is one of the first examples for accelerated approval to be granted based upon a single, albeit large, open-label, single-arm phase 1 study. Using Chambner's analogy, this is equivalent to the 3-minute mile [36, 37]. An important take-home message from the innovative certinib clinical trial has been provided by Dr. Chambner “…a well-designed phase 1 trial, even if it requires the participation of multiple institutions, can readily attract sufficient patients with uncommon tumors to prove efficacy and safety sufficient for accelerated approval.”

Taken collectively, these studies have innovated the oncology drug development paradigm by establishing the following facts:

- Phase I is not exclusively about safety and dose
- Conditional marketing approvals can be obtained from well-designed phase 1 and phase 2 studies
- Postapproval phase 3/4 commitments can provide additional information about optimizing the use of the drug and facilitate identification of safety signals missed in smaller patient populations

Despite the major benefits afforded patients for early access to innovative drugs, accelerated approvals are not without concerns. There is an expanding number of examples of accelerated approvals granted based upon uncontrolled trials using no comparison to the standard of care. Although beneficial in bringing new medicines to patients quicker and cheaper, there are, of course, significant concerns in single arm, uncontrolled trials related to patient selection bias and use of historical control data to assess clinical value. Most importantly, without patient randomization into a treatment arm and control arm, it is difficult to distinguish if the therapy targeted to a marker lesion is active and predictive of response or is the marker just a prognostic indicator.

### 3.3. Approval for Indications That Are Tissue and Site Agnostic

Historically, drug approvals were based upon antitumor efficacy in a specific tumor type for a specific line of therapy compared to some control treatment or supportive care. In the last decade, many targeted therapies have approved that target lesions present in a diverse range of tumor histologies. An example is vemurafenib, a B-Raf enzyme inhibitor selective and specific for B-Raf genes that contain a V600E or V600K BRAF mutation [45]. These mutations code for an enzyme that is constitutively activated for growth signaling properties. If a tumor contains this B-Raf V600E mutation, vemurafenib can inhibit the B-Raf/MEK/ERK pathway, which may then lead to programmed cell death. Importantly, approximately 60% of melanomas have this specific B-RAF mutation. Melanoma cells that do NOT contain these mutations are not inhibited by vemurafenib.

Vemurafenib received FDA and European Commission approval for the treatment of melanoma in August 2011, and February 2012, respectively. Vemurafenib is indicated for the treatment of patients with unresectable or metastatic melanoma with a BRAF V600E mutation, as detected by an FDA-approved test. Approval was predominantly based upon the BRIM3 trial, a phase 3 trial conducted in previously untreated melanoma patients who were randomized one-to-one between vemurafenib or the control treatment of dacarbazine, which at the time was the “standard of care” drug for the treatment of metastatic melanoma.

Mutations in B-Raf have been found in about 8% of all tumors, including non-Hodgkin lymphoma, colorectal cancer, prostate, ovarian, biliary tract, papillary thyroid carcinoma, hairy cell leukemia, non-small-cell lung carcinoma, and some glioblastomas and astrocytomas [45–47]. Each of these different cancers have very different standard of care treatment regimens which are associated with different efficacy and safety profiles. Traditionally, each would require a separate regulatory approval pathway to demonstrate clinical value compared to the currently accepted standard of care treatment regimens, despite harboring a B-RAF mutational lesion, Hence, blanket approval for vemurafenib across all tumor indications harboring a B-RAF mutation was not reasonable, since a randomized trial against standard of care would be required for each indication to demonstrate clinical value.

In November 2017, the FDA also granted regulatory approval for vemurafenib for the treatment of patients with Erdheim–Chester Disease (ECD) with BRAF V600 mutation. Approval was based on an open-label, multicenter, single-arm, multiple cohort clinical trial in 22 patients with BRAF V600 mutation-positive ECD. This approval was based upon a distinct application, despite vemurafenib targeting the exact molecular lesion as was in the melanoma trial.

Innovation and flexibility of the FDA were demonstrated on May 23, 2017, when the FDA granted accelerated approval for the first tissue/site indication agnostic approval of a drug for solid tumors who harbor specific genetic lesions, regardless of the tissue of origin or site of the tumor. The drug was pembrolizumab, and the genetic lesions were the presence of a deficiency of mismatch repair (dMMR) or microsatellite instability-high (MSI-H). This was the first FDA approval of a cancer treatment based entirely on the presence of genetic lesions rather than a specific cancer type indication [48–55].

Pembrolizumab targets the programmed cell death (PD-1) receptor found on T cells. PD-1 binds to PD-L1, a protein found on normal cells. This PD-1/PD-L1 interactions normally acts as a type of “off switch” that prevents T cells from attacking normal cells in the body. However, some cancer cells may also express PD-L1, enabling those cancer cells to evade immune recognition and destruction. Cancer therapies that block PD-1/PD-L1 interactions may allow immune recognition of transformation-associated antigens expressed on tumors. The greater the magnitude of transformation-associated antigens on the tumor, the greater the probability of efficacy by these checkpoint inhibitors.

Mismatch repair (MMR) is a cellular process that can recognize and repair erroneous insertions, deletions, and mis-incorporations of bases into DNA that can arise either during DNA replication or DNA repair of damage. The scientific rationale behind the pembrolizumab tissue agnostic approval is that tumors deficient in the MMR process have a significantly increased amount of somatic mutations. These somatic mutations have a high potential to encode and generate immunogenic antigens resulting in a higher response to immune checkpoint blockade. Hence dMMR-associated tumors may be more responsive to therapies, like pembrolizumab, that stimulate an immunological tumor response. Supportive of this rationale is that pembrolizumab activity is increased if the patients' tumors have a higher mutational burden resulting from dMMR and MSI-H.

The FDA approval of pembrolizumab in dMMR or MSH-H was based upon the combined results of 5 single-arm clinical studies in a total of 149 patients. What is so innovative about this approach is that the study included any patient who had unresectable disease, had previously received 2 or more cancer treatments, and expressed a dMMR or MSH-H tumor phenotype, regardless of the tumor type or histology. In fact, 15 different types of cancers were evaluated in these 149 patients. More specifically, ninety patients from the total 149 had CRC, while the 59 other enrolled patients presented with 14 other cancer types. Taken collectively, the results demonstrated that 60/149 patients (40%) with MSH-H or dMMR tumors had very significant tumor reductions. Very significantly, in one of those 5 trails, 40% of colorectal patients that had dMMR tumors responded to pembrolizumab treatment but 0% of colorectal patients that had MMR proficient tumors responded to pembrolizumab treatment.

The enabling factors that help realize this tissue agnostic approval were as follows:

1. A deep understanding of the cellular mechanism of pembrolizumab
2. Strong scientific rationale between the mechanistic association between PD-1/PD-L1 inhibition and tumors that have the dMMR or MSH-H phenotype
3. Patient enrollment with advanced disease with little to no alternative treatment options
4. Finally, FDA-accelerated approval granted contingent upon a postmarketing commitment to validate and further define the clinical benefit with other therapies in colorectal cancer patients that have dMMR or MSI-H tumors.

### 3.4. Integration of Clinical Trials as a Clinical Care Option

Comprehensive clinical management has routinely comprised the integration of medical, surgical, and radiation oncologists plus supportive staff. Clinical trial participation was not routinely viewed as a critical component of comprehensive clinical care. Participation was encouraged in two settings:

1. Encouraged as an option in a salvage therapy situation when all approved treatments have failed
2. When the treating physician was also participating in an ongoing trial

With the current advances in Precision Medicine utilizing targeted- and immune-therapies, it is now appreciated that patients randomized into early and late stage trials now have the opportunity to

1. Experience significant clinical benefit in these targeted trials that select subpopulations of patients that will benefit the most
2. Have the opportunity to be treated with cutting edge and innovative medicines that may have significant clinical value over the current standard of care drugs.
3. Using innovative trail designs employing surrogate markers can be timely switched to the standard of care medicines if the investigational drug appears to have little clinical benefit
4. To receive robust medical care including pain management in a clinical trial setting

As such, participation in early- and late-stage clinical trials is becoming a routine and important care option, not restricted to the salvage therapy. It is important to note that, in 2013, less than 1% of the US population participated in clinical trials, yet 72% say they would participate if it was recommended by their physician [56].

### 3.5. Clinical Trials and Real-World Evidence: Necessity for Both

There will always be the need for highly controlled, randomized clinical trials. This review highlights some of the innovative approaches being utilized in the oncology clinical trial setting to help drive effectiveness and efficiency. However, it must be appreciated that, despite how well designed and robust a clinical trial is, it is not meant to answer all the critical questions regarding a new oncology drug. It is appreciated that there may be significant differences in clinical outcomes comparing how a drug performs in a well-controlled trial compared to how a drug performs in routine clinical practice, i.e., in the “real-world.” An NDA data package compile comprehensive, well controlled, data essential for the FDA and other regulatory review bodies to assess authorization for marketing; yet it may be incomplete for other key stakeholders, such as 3^rd^ party payers and some needs of patients and prescribers. At the time of NDA submission, real-world evidence (RWE) may be significantly lacking to answer fundamental questions of activity in routine clinical practice and pharmacoeconomic value relative to other treatment options. RWE clearly impacts optimal clinical practice and reimbursement strategy, as the data are generated from use in routine oncology practices in the real world. It is recognized that RWE data have routinely impacted postmarking activity involving indication expansion, market access, formulary decisions, and 3^rd^ party payer reimbursement decisions. However, RWE can also significantly inform pre-NDA clinical development strategy. RWE has promised to help shorten clinical development time-lines and potential to help both clinical success and commercial success [57–59]. Some applications of RWE in the pre-NDA setting are helping to optimally design a clinical protocol to take into account such real-world data as follows:

- How standard of care is used in the real-world
- Use of historical or contemporaneous standard of care control arms
- Inclusive/exclusion criteria—small changes may make a huge impact on accrual
- Details of clinical assessments in real-world clinical practices

### 3.6. Pay for Performance and Value-Based Pricing

Prior to marketing approval, while drugs are in clinical evaluation trials, there is a significant incentive for biopharmaceutical companies to objectively design their trials to enroll “the right patients at the right dose.” Hence, the objective in trial design is to objectively and transparently select and enrichment for patients who will more optimally respond to their drug, via protocol inclusion and exclusion criteria, to obtain better clinical outcomes. Better clinical outcomes via thoughtful and objective patient enrichment strategies will, of course, result in higher probability of marketing approval. It is also highly likely that the patient enrichment/selection strategy used in the clinical trials will also become part of the prescribing label for the approved use of the product. Although the approved use may be restricted to specific patient populations, optimal clinical outcomes in this restricted patient population may also be associated with the opportunity for premium pricing due to better clinical outcomes.

With the growing pressures on oncology drug prices, it is imperative that new financial models must evolve to ensure both drug access and pharmacoeconomic value creation [60]. There is a delicate balance between enabling patient access to potentially clinically beneficial new oncology medicines and financial determinants for reimbursements via third party payers. Third party payers have a clear incentive to evolve reimbursement mechanisms that help control costs without withholding potentially beneficial new medicines from patients [61]. However, it must be recognized that a simple focus on cost is too simplistic. If cost alone was the focus, then pricing and discounts would be the simple solution. A major driver for cost containment should focus on ensuring the right oncology drug is selectively used in the right patient population, while incentivizing that the drug is not used in patients with little hope of clinical benefit.

#### 3.6.1. Shift from Volume to Value

Traditionally, biopharmaceutical companies got paid based upon units sold. Once regulatory approval for marketing is obtained, revenue is predominantly based upon volume, not how well the drug works in the indicated patient population in a real-world setting. More and more biopharmaceutical companies and payers are now partnering to make drug cost reimbursements predicated on drug performance based upon a prospectively agreed clinical metrics. This shift is transitioning from a *Volume Metric to a Value Metric*, based upon clinical outcomes [62, 63]. In many cases, there are agreements between third party insures with drug companies that the drug company will be reimbursed at a set price, which provided the drug meets or exceeds a clinical value metric. This *Pay for Performance* financial model has many different terms describing it: *Pay for Performance (P4P)*; *Value-Based Pricing*; *Clinical/Financial Risk-Sharing*; *Performance-Based Reimbursement*; *Outcome-Based Risk-Sharing Agreements (OBRAs).*

The P4P financial model is especially relevant for oncology drugs for the following reasons:

1. New and innovative oncology drugs have been very highly priced, some of which are over $100,000 per patient per year. In many cases, the price is related more to what the market will bear rather than value.
2. There has been a tremendous advance in the basic cellular and molecular understanding of cancer resulting in many new oncology drugs being either specific targeted therapies or immunotherapeutic. This has fostered the intent to selectively treat the right subpopulation of patients that display the correct genotypic and phenotypic signatures for that targeted agent. However, there is still a wide breadth of clinical outcomes in patients, despite their genetic and biomarker signatures due to complexity and heterogeneity.
3. Clinical value in a real-world setting may be different than in a highly controlled clinical trial setting.
4. Throughout an oncology drug's lifecycle, a drug is likely to receive regulatory approval in multiple oncology indications. It is also likely that this drug will exhibit a wide range of clinical benefit depending upon those different indications. Yet there will be no differential drug pricing for indication.

#### 3.6.2. Benefits of P4P

In the simplest terms of P4P, payers only pay for the drug if a prospectively agreed patient benefit is realized. P4P greatly incentivizes drug makers to

1. Focus on medical value not volume
2. Invest in identification of prognostic indicators to prospectively help identify responders and nonresponders
3. Extend effort to ensure the health care professionals are using drug in the right patient population.
4. Focus on methods to encourage compliance, at both the physician and patient levels

Bortezomib (Velcade), a proteasome inhibitor drug, was approved in the U.S. and Europe for treating multiple myeloma. The National Institute for Health and Care Excellence (NICE) initially recommended against Velcade in October 2006 because NICE reported that the date did not demonstrate sufficient cost-effectiveness of the drug, despite extending the life expectancy by an average of six months over the standard treatment [64]. NICE stated *“Although the drug is clinically effective compared with high-dose dexamethasone, its cost-effectiveness has not been satisfactorily demonstrated and therefore further research is required.”* NICE assed the cost-effectiveness of Velcade by analyzing the cost and benefit relative to dexamethasone, the next best treatment.

In response, the pharmaceutical company chose not to simply reduce the drug cost to achieve cost-effectiveness of Velcade, but rather proposed a performance-linked cost reduction scheme for patients with multiple myeloma, and this was accepted [64, 65]. The pharmaceutical company essential proposed to charge only when the drug was effective and to refund the drug costs if it was not clinically effective. Clinical response to Velcade was defined as those patients achieving a 25% or greater reduction in serum M-protein within the first 4 cycles of treatment. If the treatment did not achieve a 25% or greater reduction of serum M-protein, then the pharmaceutical company would reimburse the NHS for the cost of the first four cycles. This innovative financial risk share gives the pharmaceutical company strong incentive to maximize the number of patients who respond through personalized selection process, rather than simple maximized number of treated patients [66].

## Figures and Tables

**Table 1:** Illustration of new cancer diagnosis and 5-year survival rates of different cancer types in the US [1].

Cancer type	Estimated new cases in 2016	Estimated 5-year relative survival rate in 2016
Testis	8,720	95%
Thyroid	64,300	98%
Melanoma	76,380	98% if localized; 17% if advanced

Pancreas	53,070	7%
Liver and bile duct	39,230	17%
Lung and bronchus	224,390	17%
Esophagus	16,910	18%
Gallbladder	11,420	18%
Gastric	26,370	26%
Acute myeloid leukemia	19,950	26%
Brain	23,770	29%

**Table 2:** Median yearly cost in the US of 51 new oncology drugs approved between 2009 and 2014 [3].

Total oncology drugs approved by the FDA from 2009 to 2014	Primary endpoint for approval (% of total; *N*)	Median price per year of treatment
51	ORR (35%; 18)	$137,952
	PFS (35%; 18)	$102,677
	OS (30%; 15)	$112,370

ORR: objective response rate. PFS: progression fee survival. OS: overall survival (table adapted from [3]).
